# Skin lesions in patients treated with growth hormone and those with growth hormone excess: a current overview

**DOI:** 10.3389/fmed.2026.1777658

**Published:** 2026-03-12

**Authors:** Mateusz Matwiejuk, Hanna Myśliwiec, Agnieszka Miklosz, Adrian Chabowski, Iwona Flisiak

**Affiliations:** 1 Department of Dermatology and Venereology, Medical University of Bialystok, Bialystok, Poland; 2 Department of Physiology, Medical University of Bialystok, Bialystok, Poland

**Keywords:** acromegaly, carney complex, gigantism, growth hormone, growth hormone therapy, McCune-Albright syndrome, multiple endocrine neoplasia type 1, neurofibromatosis

## Abstract

Growth hormone (GH) is an ancestral hormone secreted from the anterior pituitary gland. In adulthood, it is essential to regulate metabolism. GH synthesis and secretion are regulated in a complex manner, primarily through the actions of hypothalamic neuropeptides (GHRH and somatostatin) that integrate hormonal, metabolic, and neurogenic signals. Currently, recombinant human GH is widely used to treat growth hormone deficiency (GHD) and numerous non-GHD disorders, such as short stature and catabolic conditions. Conversely, an excess of GH may lead to different and severe conditions, such as acromegaly, gigantism, Carney complex, McCune–Albright syndrome, neurofibromatosis, and multiple endocrine neoplasia type 1. In patients with growth hormone excess disorders or those treated with GH, skin manifestations are common and can include skin thickening, coarsened facial features, skin tags, oily skin, and excessive sweating. These dermatological changes result from the direct actions of GH and IGF-1 (insulin-like growth factor 1) on skin cells and appendages, leading to increased collagen synthesis and connective tissue expansion. This review focuses on the various skin symptoms associated with these disorders caused by GH excess. This narrative review summarizes recent findings on the management of skin lesions in GH-treated patients and in those with GH excess, highlighting the benefits, side effects, and limitations of current therapies.

## 1 Introduction

### 1.1 Growth hormone (GH) physiology

Growth hormone (GH) is a single-chain polypeptide composed of 191 amino acids and 2 disulfide bonds (1). Its secretion from the somatotropic cells of the anterior pituitary gland is highly pulsatile, with approximately 70% of the daily large-burst secretion occurring during the night. This sleep-dependent secretion is primarily driven by the hypothalamic release of growth hormone-releasing hormone (GHRH) and growth hormone-inhibiting hormone (GHIH) (2). GH is an anabolic hormone that promotes protein synthesis, lipolysis, and growth. Before puberty, GH mainly boosts skeletal and lean body mass (LBM) growth and its differentiation. As individuals mature, GH’s role shifts toward the regulation and maintenance of body composition (3).

### 1.2 Growth hormone deficiency (GHD)

Growth hormone deficiency (GHD) is a condition characterized by insufficient production of growth hormone. It can be isolated, occurring as the sole pituitary hormone deficiency, or it may coexist with deficiencies in other pituitary hormones, a condition known as hypopituitarism or multiple pituitary hormone deficiency (MPHD) (4). Children whose height falls below the third percentile or −2 standard deviations (SDs) for their age and sex should be evaluated for short stature (5). Childhood-onset growth hormone deficiency (CO-GHD) is estimated to occur in 1 per 30,000 children annually, whereas adult-onset growth hormone deficiency (AO-GHD) occurs in approximately 1.2 per 100,000 adults (6). CO-GHD can be idiopathic, with no identifiable cause, or may result from congenital, structural, or acquired factors (7). In contrast, AO-GHD is frequently acquired and often associated with hypothalamic–pituitary tumors or their treatment (8). Growth hormone replacement therapy is highly effective in treating children with GHD, with the primary goal of normalizing adult height. In addition, it is a highly recommended and well-established approach for addressing metabolic and psychological abnormalities associated with GHD (9).

### 1.3 Growth hormone excess

Growth hormone excess is a rare and usually benign condition, typically caused by a pituitary tumor leading to excessive production of GH and IGF-1. In adults, this results in acromegaly, characterized by enlarged bones, hands, feet, and soft tissues, often resulting in facial feature coarsening. If it occurs in children before the growth plates fuse, it causes gigantism, resulting in excessive height.

### 1.4 Skin manifestations of growth hormone-induced diseases

Growth hormone exerts significant effects on various skin cell types, with GH receptors present in the majority of skin cells, whereas IGF-1 receptor expression is largely restricted to epidermal keratinocytes. Excess GH affects the skin, leading to a thicker, coarser texture due to increased collagen production in the dermis. Furthermore, excess GH is associated with hypertrichosis (excessive hair growth), hyperhidrosis (excessive sweating), cutis verticis gyrata (scalp folds resembling the gyri of the brain), and increased sebum production. Some patients also develop acne-like lesions, perifollicular pigmentation, and acanthosis nigricans. Oversecretion of GH leads to the accumulation of glycosaminoglycans (GAGs), particularly hyaluronic acid, chondroitin sulfate, and dermatan sulfate within the dermal tissue. GAGs are highly hydrophilic (water-attracting), so their accumulation results in water retention and edema in the interstitial space of the skin. This causes structural changes, producing a doughy, swollen, and thickened skin texture, most pronounced in the face, hands, and feet (10). Importantly, skin manifestations are crucial diagnostic clues and often accompany specific growth hormone excess disorders. Neurofibromatosis presents with classic café-au-lait spots, while McCune–Albright syndrome is characterized by larger, jagged-edged, irregular hyperpigmented regions, aiding in distinguishing these GH-induced disorders. Meticulous dermatological examination can aid in identifying the underlying condition. Conversely, GH deficiency is characterized by reduced skin capacitance and sebum content, leading to dry, thin, and sometimes wrinkled skin, as well as decreased sweat gland function.

### 1.5 Unmet needs in dermatology and endocrinology

Unmet needs in dermatology and endocrinology often intersect, particularly regarding chronic inflammatory conditions, autoimmune diseases, and the systemic effects of hormonal imbalances on the skin. While both fields have advanced with targeted therapies, significant gaps remain in early diagnosis, personalized treatment, and the management of long-term side effects. Recently, hyperspectral imaging (HSI) combined with computer-aided diagnosis (CAD) systems has emerged as a state-of-the-art, non-invasive technology for the early, precise, and objective detection of skin diseases, often surpassing the limitations of traditional, subjective, and invasive biopsy methods. Hyperspectral imaging, characterized by its exceptional ability to capture information across a wide range of the electromagnetic spectrum, surpasses conventional red–green–blue (RGB) imaging that relies on only three colour channels. This enables the extraction of detailed spectral “fingerprints” that reveal tissue composition, oxygenation, and subtle biochemical changes. Therefore, HSI can be used as a diagnostic modality for the early identification of skin cancer, with the goal of improving therapeutic outcomes and advancing the technical capabilities in the field of skin cancer detection (11).

In another study, the HSI–based Spectrum-Aided Vision Enhancer (SAVE) method provided dermatologists with a tool for early and accurate diagnosis, reducing the likelihood of misclassification and improving patient outcomes. HSI, when combined with machine learning (ML) or deep learning models, increases diagnostic sensitivity and specificity, for instance, by significantly reducing misclassification of malignant, pre-malignant (e.g., actinic keratosis), and benign lesions. SAVE is a novel approach that transforms standard RGB images into narrow-band images (NBI) by integrating HSI techniques to boost the differences between cancerous lesions and surrounding normal tissue. The researchers reported that the SAVE method significantly improved diagnostic accuracy, sensitivity, and specificity compared with conventional RGB imaging, demonstrating its potential to enhance the performance of skin cancer classification algorithms (12). These advancements are critical for the early identification of melanoma and other skin cancers, which is vital for improving patient survival rates and reducing the need for unnecessary biopsies.

The review summarizes the available data on skin lesions in patients treated with growth hormone and in growth hormone excess disorders, providing an overview of the current state-of-the-art.

## 2 Methods

The search resulted in the screening of 105 records, of which 61 were assessed for eligibility, and 43 were ultimately included in the qualitative synthesis.

A medical literature search of PubMed (1937–present), Google Scholar, and Embase, conducted in the spring of 2025, was performed using appropriate terms without date limitations. The main objective of the research was to identify the association between growth hormone therapy and skin lesions. Medical subject headline terms included “acromegaly and skin lesions,” “neurofibromatosis and skin lesions,” “McCune–Albright syndrome and skin lesions,” “multiple endocrine neoplasia type 1 and skin lesions,” “Carney complex and skin lesions,” “Sheehan’s syndrome and skin lesions,” “growth hormone and skin diseases,” “growth hormone therapy and skin diseases,” “growth hormone therapy and adverse effects,” and “growth hormone therapy and skin lesions.”

Non-English publications, articles with low clinical relevance (which did not reveal significant outcomes), articles written in a language other than English, and duplicated publications were excluded from the analysis. Originally, human and animal studies were included in this narrative review. The results of the search strings were combined, and duplicates were removed. Afterwards, the titles and abstracts of the searched studies were independently screened by two reviewers (M.M. and H.M.) to identify relevant articles that addressed the review subject. Disagreements between reviewers were resolved by a fourth reviewer (A.C.). Finally, the selected eligible articles were fully reviewed.

## 3 Discussion

### 3.1 The physiological impact of GH on skin

#### 3.1.1 Growth hormone effect on fibroblasts

Growth hormone stimulates fibroblast activity, primarily enhancing their proliferation, migration, and differentiation, which accelerates wound healing and collagen synthesis. It promotes these actions by increasing local IGF-1 production, activating the ERK signaling pathway, and boosting collagens type I and type III synthesis (13).

Azimi et al. (13) studied the impact of the transferosome-delivered human GH on dermal fibroblast cells. They found that transdermal delivery of encapsulated human growth hormone (hGH) can boost the proliferation, migration, and gene expression of collagen types I and III (14). Specifically, the authors examined the ability of two transferosome formulations (F1 and F2) to deliver GH through the skin over 24 h. The maximum amount of GH that penetrated the skin was 489.54 ng/cm^3^ for F1 transferosome and 248.46 ng/cm^3^ for F2 transferosome. The researchers found that the F1 formulation was more efficient, based on the higher amount of GH delivered, and showed no toxicity against cells. Taken together, GH can influence fibroblast growth and division, which are crucial for collagen synthesis, skin aging, and wound healing (13) (Table 1).

**Table 1:** Summary of the studies on the effect of the human growth hormone.

Author	Year	Population	Key observation
The effect of the human growth hormone			
Azimi et al. (13)	2019	Fibroblasts	hGH can boost the proliferation, migration, and expression of collagen types I and III.
Skin thickness			
Rudman et al. (15)	1990	21 healthy people	Reduced GH secretion in older age results in decreased lean body mass, increased adipose tissue mass, and thinning of the skin.
Kann et al. (16)	1996	20 patients with growth hormone deficiency	GH administration increased collagen type I production, resulting in an accumulation of collagen type I in the skin.
Lonn et al. (17)	1996	10 patients with pituitary deficiencies	Preferential lipid mobilisation, which occurred in the visceral and subcutaneous trunk depots, led to an altered adipose tissue distribution.
GH effect on scar formation			
de Oliveira et al. (18)	2004	62 patients	Collagen types I and III were elevated, statistically significantly, in the reticular layer of scar in comparison to healthy skin. rhGH did not cause a negative effect on scarring formation.
Handler et al. (19)	2012	49-year-old patient diagnosed with melanoma; 51-year-old wife also diagnosed with melanoma	Melanoma developed in two unrelated individuals after they both used GH. GH might have growth-promoting effects on melanomas.
Kędzia et al. (20)	2014	15-year old girl with Turner syndrome	There might be a link between hypertrophic scars and keloids during rhGH therapy.

hGH, human growth hormone; IGF-1, insulin growth factor 1; rhGH, recombinant human growth hormone.

#### 3.1.2 Growth hormone effect on the skin thickness

GH increases skin thickness by stimulating collagen type I synthesis, boosting elastin production, and promoting dermal fibroblast proliferation via IGF-1. It enhances skin density, improves tensile strength, and increases hydration. Conversely, GH deficiency results in thin, dry, and fragile skin, while excessive GH can lead to skin thickening. Rudman et al. (15) studied the activity of the GH/IGF-1 axis in men over 60 years old. Twenty-one healthy men (61–81 years old) with low baseline IGF-I (<350 U/L) were divided into a treatment group (12 men) and a control group (9 men) over 6 months. Participants received 0.03 mg of biosynthetic human GH per kilogram of body weight subcutaneously three times a week. This resulted in their mean plasma IGF-I levels rising into the “youthful” range of 500 to 1,500 U/L. In the group without treatment, IGF-I levels remained below 350 U/L, with no significant changes in body composition, adipose-tissue mass, or bone density. Furthermore, in the treatment group, due to GH implementation, lean body mass increased by 8.8%, adipose-tissue mass decreased by 14.4%, median lumbar vertebral bone density increased by 1.6%, and finally, skin thickness increased by 7.1%. The declining activity of the GH/IGF-1 axis with advancing age may contribute to the decrease in lean body mass and the increase in mass of adipose tissue that occur with aging (15) (Table 1).

Similar to the abovementioned study, Kann et al. (16) observed that growth hormone application (dose of 0.25 U/kg/week) can lead to an increase in skin thickness by boosting the synthesis of collagen type I (16) (Table 1).

Lonn et al. (17) also demonstrated that GH increased the thickness of the combined musculocutaneous compartment by 2.4% (*p* < 0.05). However, it is unclear to what extent this increase is due to skin growth or specifically muscle growth. Therefore, determining the exact contribution of each type of growth to overall size increase can be complex (17) (Table 1).

#### 3.1.3 Growth hormone on scar formation

de Oliveira et al. (18) found that recombinant human growth hormone (rhGH) in severely burned children did not negatively impact scarring. Both the rhGH group and the control group showed increased levels of collagen types I and III in scar tissue compared to normal skin, indicating that scarring is a natural response to injury, regardless of growth hormone treatment. The rhGH group did exhibit significantly higher levels of IGF-1, but there were no observable differences in scar appearance, size, or other characteristics between the two groups, suggesting that rhGH treatment did not worsen scarring (18) (Table 1).

Handler et al. (19) showed that hGH can promote melanoma growth, potentially worsening the condition. This effect is thought to be mediated by direct stimulation of hGH receptors, which upregulate the MAPK/ERK pathway. The pathway is involved in cell growth and proliferation, which emphasizes the role of hGH in promoting mitogenic signaling in melanoma. The study also highlighted the increasing use of hGH, particularly for anti-aging purposes (19) (Table 1).

Kędzia et al. (20) suggested a possible link between rhGH therapy and an increased risk of hypertrophic scars and keloids in Turner’s syndrome (TS) patients undergoing surgical procedures. The concern stems from the possibility of excessive IGF-I levels due to the combination of rhGH and endogenous GH (20) (Table 1).

### 3.2 Skin manifestation in GH-related disorders

#### 3.2.1 Acromegaly

Acromegaly is a chronic and progressive condition characterized by GH excess and elevated IGF-1 levels, attributed in the vast majority of cases to a benign pituitary adenoma. Acromegaly is a rare endocrine disorder with an estimated prevalence of approximately 2.8 to 13.7 cases per 100,000 people and an annual incidence of approximately 0.2 to 1.1 new cases per 100,000 individuals. The condition affects men and women with similar frequency, and the median age at diagnosis is typically in the fourth to fifth decade of life, most often around 40–45 years. If left untreated, acromegaly is associated with substantially increased mortality—historically reported up to two to three times higher than in the general population, primarily due to cardiovascular and respiratory complications—although effective treatment can normalize survival (21).

Degirmentepe et al. (22) described the case of a 37-year-old patient suffering from acromegaly who was diagnosed with soft tissue hyperplasia and skin thickening. The patient had numerous pigmented skin tags on the neck, which is a common finding in individuals with acromegaly. The scalp thickening and multiple, gyriform skin folds, particularly in the occipital region, are known cutaneous manifestations associated with acromegaly. Additionally, the velvety brownish-dark plaques and soft, brown hyperpigmented polypoid growths spotted on the neck, groin, and bilateral axillae confirm the presence of acanthosis nigricans, alongside acromegaly (22) (Table 2).

**Table 2:** Summary of the studies on acromegaly and syndromes related to those diseases.

Author	Year	Population	Key observation
Acromegaly			
Degirmentepe et al. (22)	2017	n/a	Acromegaly is known for soft tissue overgrowth and skin thickening, oily skin with enlarged pores, hypertrichosis, excessive sweating, pigmented skin tags, acanthosis nigricans, and psoriasis, which are frequently observed.
Resende et al. (23)	2012	15 patients dealing witch acromegaly	Acromegaly is characterized by multiple skin symptoms, skin thickening, acrochordons, epidermoid cysts, pseudoacanthosis nigricans, seborrheic keratoses, melanocytic nevi, and lentiginous spots.
Borlu et al. (24)	2012	24 healthy people 52 patients with acromegaly	GH and IGF-1 were positively correlated with sebum levels and negatively correlated with skin temperature on the forehead and the forearm.
Krsek et al. (25)	2002	15 healthy people 13 patients with acromegaly	Serum levels of IGFBP-1, total cholesterol, and LDL cholesterol in the group of patients with hormonally active acromegaly.
Neurofibromatosis			
Nunley et al. (26)	2009	110 patients with caffe-au-lait; 34 patients with NF	Skin lesions in NF-1 are café-au-lait spots, axillary or inguinal freckling, Lisch nodules, neurofibromas, skeletal anomalies, macrocephaly, optic gliomas, plexiform neurofibromas, and precocious puberty.
Crowe et al. (28)	1964	223 patients with NF-1; 45 patients with NF-1 and axillary freckling	Axillary or inguinal freckling is a significant indicator for NF-1 diagnosis.
Carney complex			
Stratakis et al. (34)	1996	101 all patients included in the study Including 50 patients with Carney complex	Carney complex is characterized by multiple nevi. Potty skin pigmentation.
Sheehan’s syndrome			
Borlu et al. (37)	2007	20 healthy people 21 patients with Sheehan’s syndrome	Sheehan’s syndrome is characterized by decreased sebum content and skin capacitance on the forearm and forehead.

GH, growth hormone; IGF-1, insulin-like growth factor-1; LDL-cholesterol, low-density lipoprotein cholesterol; TEWL, transepidermal water loss; IGFBP-1, insulin-like growth factor-binding protein 1; NF-1, neurofibromatosis-1.

Resende et al. (23) reported that all patients with acromegaly in their study exhibited various dermatological manifestations, including skin thickening, acrochordons (skin tags), epidermoid cysts, pseudoacanthosis nigricans, seborrheic keratosis, melanocytic nevi (moles), and lentiginous spots. The primary issue of skin lesions stems from an excess of GH and insulin growth factor (IGF-1), which have potent trophic and metabolic effects on various tissues, including the skin. Precisely, histological examination reveals deposits of GAGs, particularly hyaluronic acid and chondroitin sulfate, in the reticular layer of the dermis. This leads to increased water retention, causing skin thickening and edema. Additionally, a high amount of melanotropin hormone leads to hyperpigmentation. Furthermore, GH impacts testosterone levels by decreasing sex hormone-binding globulin (SHBG), resulting in higher free testosterone and potentially hirsutism. Finally, GH and IGF-1 stimulate sebaceous glands, causing oily skin and enlarged pores (23) (Table 2).

Borlu et al. (24) reported that in patients with acromegaly, the skin has more oil (sebum) and a more alkaline pH compared to healthy subjects. Baseline GH and IGF-1 levels, as well as levels after 3 and 6 months of treatment, were positively correlated with sebum levels on the forehead and forearm in the studied patients. This indicates that higher GH/IGF-1 levels are associated with increased sebum production and lower skin temperature. Interestingly, GH and IGF-1 levels were negatively correlated with skin temperature on both the forehead and forearm. In addition, an increased pH, decreased transepidermal water loss (TEWL), and lower skin temperature are likely related to excessive perspiration, which is attributed to GH’s direct influence on sweat glands (24) (Table 2).

Acromegaly is associated with impaired skin microcirculation, specifically a reduced ability of small blood vessels to increase blood flow after occlusion or heating, as shown in a study by Krsek et al. (25). The authors used laser Doppler to measure blood flow in patients with acromegaly. The researchers revealed a significant decrease in maximal perfusion after occlusion (PORHmax) and after heating (THmax); moreover, an increase in blood flow velocity was observed after both occlusion and heating. This suggests that elevated GH and IGF-1 in acromegaly are associated with impaired microvascular function (25) (Table 1).

Higher serum-free IGF-1 levels correlate positively with a longer time to peak blood flow after arterial occlusion (*r* = 0.55, *p* < 0.01), suggesting a slower reactive hyperemia response (25). Concurrently, increased carotid intima-media thickness shows a negative correlation with peak blood flow after arterial occlusion (*r* = −0.66, p < 0.01), indicating impaired vascular function and potentially compromised delivery of oxygen and nutrients to tissues [248] (Table 2).

#### 3.2.2 Neurofibromatosis type 1 (NF1)

Neurofibromatosis type 1 (NF1) is an autosomal dominant disorder caused by loss-of-function mutations in the *neurofibromin 1* (*NF1*) gene on chromosome 17q11.2. Approximately 50% of cases are inherited, while 50% result from *de novo* mutations. These mutations reduce the activity of the tumor suppressor protein neurofibromin, causing unregulated cell growth. Neurofibromatosis type 1 is frequently associated with GH axis abnormalities, including both deficiency (GHD) and, more rarely, excess (26). Children with NF1 may experience short stature due to suprasellar lesions or idiopathic GHD (27). While GH treatment for deficiency is generally considered safe and beneficial, the role of GH in potentially stimulating neurofibroma growth remains a subject of research (14).

Nunley et al. (26) described that café-au-lait spots are light brown, flat birthmarks that are a hallmark of neurofibromatosis type 1 (NF-1) syndrome. In this study, axillary or inguinal freckling was the most frequently observed second diagnostic feature of NF, appearing in 26 out of 34 patients (77%). Other signs that served as the second diagnostic feature, though less common, included: Lisch nodules, plexiform neurofibroma, and tibial pseudarthrosis (26) (Table 2).

The study by Crowe et al. (28) highlighted that axillary or inguinal freckling is a significant indicator for NF-1 diagnosis, potentially affecting up to 80% of patients. This adds another layer to diagnosing NF-1 beyond just cafe-au-lait spots (28) (Table 2).

#### 3.2.3 McCune–Albright Syndrome (MAS)

McCune–Albright Syndrome (MAS) commonly features excess GH production, occurring in up to 21–30% of cases due to somatic *GNAS1* mutations. This often leads to pituitary adenomas, causing accelerated growth, acromegaly, or gigantism, commonly in conjunction with precocious puberty. MAS is considered an ultra-rare disease, and its prevalence is estimated between 1 in 100,000 and 1 in 1,000,000 worldwide. The condition affects both men and women (29).

In McCune–Albright syndrome (30), café-au-lait macules are a common feature, affecting approximately two-thirds of patients. These pigmented skin patches can be an early indicator of the syndrome, often appearing at birth or shortly after. Histopathological examination revealed a medium-thick epidermis, a thin superficial layer of keratinized cells, granulosa cells containing moderate amounts of amber-colored pigment, and stratum germinativum cells possessing abundant similar pigment. In the dermis, only occasional pigmented macrophages were visible in the superficial layer, and hair follicles, sebaceous glands, and sweat glands were moderately numerous. Erector pili muscles were well-developed, whereas blood vessels and nerves were absent. Pigment testing revealed no iron staining, indicating the presence of melanin. Overall, skin examination findings were negative, except for melanin deposition (30).

#### 3.2.4 Multiple endocrine neoplasia type 1 (MEN1)

Multiple endocrine neoplasia type 1 (MEN1) is a rare, autosomal dominant hereditary syndrome causing tumors in endocrine glands, primarily the parathyroid, pancreatic/duodenal neuroendocrine tissues, and anterior pituitary. The prevalence is estimated at 1 to 20 per 100,000 individuals globally (31).

The study by Darling et al. (32) is a key reference for understanding the cutaneous manifestations of multiple endocrine neoplasia type 1 (MEN1). The authors noted several key skin features, including multiple angiofibromas, collagenomas, lipomas, tiny “confetti-like” hypopigmented macules, and multiple gingival papules. Among these findings, angiofibromas were particularly common, occurring in approximately 88% of patients, with half of those affected having five or more of them. The authors identified several important skin manifestations associated with MEN1, including multiple angiofibromas, collagenomas, lipomas, confetti-like hypopigmented macules, and multiple gingival papules. In common clinical findings, angiofibromas are highly prevalent, observed in 88% of patients, with 50% having five or more. Collagenomas are also common, present in 72% of patients. Other skin symptoms include café au lait macules (38%), lipomas (34%), confetti-like hypopigmented macules (6%), and multiple gingival papules (6%) (32).

#### 3.2.5 Carney complex

The precise prevalence of Carney complex is unknown, but it is estimated to include fewer than 750 individuals worldwide across all reported cases. Regarding inheritance, approximately 70% of cases are inherited in an autosomal dominant fashion, while the remaining 30% arise from *de novo* (spontaneous) mutations. At the molecular level, Carney complex involves dysregulation of the protein kinase A (PKA) signalling pathway (33).

Stratakis et al. (34) reported that Carney complex is characterized by distinctive skin pigmentation, primarily lentigines (small, dark spots) and blue nevi (benign moles with a bluish hue). These pigmented lesions, along with multiple cutaneous myxomas (benign connective tissue tumors), are the main cutaneous manifestations. Spotty skin pigmentation is the most common clinical finding, present in approximately 96% of patients, and is also seen in other syndromes such as Peutz-Jeghers syndrome and LEOPARD syndrome. This pigmentation often consists of lentigines (small, dark spots) and blue nevi. Genetic studies have identified defects responsible for Carney complex in the short arm of chromosome 2, precisely in the 2p16 region (34) (Table 2).

#### 3.2.6 Sheehan’s syndrome

The incidence of Sheehan’s syndrome is estimated to be as high as five cases per 100,000 births. The primary etiology of this disorder is ischemic necrosis of the anterior pituitary gland, resulting from severe postpartum hemorrhage and subsequent hypovolemic shock (35).

Karaca et al. (36) showed that Sheehan syndrome is a rare, serious disease that results from postpartum pituitary necrosis, leading to severe hypopituitarism. Typical clinical features of Sheehan syndrome include sparse axillary and pubic hair, atrophy of the mammary glands, fine wrinkling around the mouth and eyes, and dry, hypopigmented skin (36).

Borlu et al. (37) showed that patients with Sheehan’s syndrome exhibited lower skin capacitance and sebum content than the control group. Specifically, skin capacitance on the forehead (*p* = 0.002) and forearm (*p* = 0.030) was significantly reduced in patients with Sheehan’s syndrome. The researchers linked these skin changes to potential GH and IGF-I deficiencies associated with Sheehan’s syndrome. Additionally, it has been shown that IGF-1 may act together with growth hormone to increase collagen synthesis to a greater extent than either growth factor alone (37) (Table 2).

The skin manifestations in each of the abovementioned conditions are mentioned in Table 3.

**Table 3:** Principal cutaneous manifestations in various conditions.

Conditions	Skin manifestations
Acromegaly	Skin tags (acrochordons), thickened/oily skin (seborrhea), acne-like lesions, hyperhidrosis, and acanthosis nigricans.
Neurofibromatosis	Lisch nodules, plexiform neurofibroma, cafe-au-lait spots, and inguinal freckling
McCune–Albright syndrome	Multiple angiofibromas, collagenomas, lipomas, confetti-like hypopigmented macules, and multiple gingival papules
Multiple Endocrine Neoplasia Type 1	Collagenomas, cafe-au-lait macules, lipomas, confetti-like hypopigmented macules, and multiple gingival papules
Carney complex	Distinctive skin pigmentation, primarily lentigines, blue nevi, and cutaneous myxomas
Sheehan’s syndrome	Dry, thin, and pale skin

## 4 Adverse effects

Mehta et al. (38) revealed that the child developed an itchy rash (pruritic papules) on the chest and upper abdomen 15 min after administration of the first dose of rhGH. However, no rash developed at the injection site. The rash was treated with hydrocortisone ointment, and it gradually disappeared within 5 days. Due to concerns about an allergic reaction, rhGH treatment was discontinued. Blood tests showed a slightly elevated eosinophil count, but the results were otherwise normal. The child was diagnosed with papular atopic dermatitis, a type of eczema, not an allergy to rhGH. Subsequently, the child received a second dose of rhGH under supervision, and the rash did not recur. This suggests that the previous rash was not a severe allergic reaction. Afterward, the child continued rhGH treatment for a year without further issues and with improved growth (38).

Mann et al. (39) reported that a 65-year-old patient receiving GH therapy developed an itchy rash with red bumps that grew into patches. The rash spread from the torso to the armpits and groin, causing damage to the superficial layer of the skin. The described skin condition was characterized by blisters resembling those caused by medications but without affecting internal mucosal linings, and showed a positive Nikolsky sign (39).

## 5 Limitations

Many studies have utilized small, uncontrolled samples, limiting the statistical power and generalizability of findings. Moreover, there is a lack of consistent, standardized dermatological assessment methods for monitoring skin lesions in patients receiving GH. Importantly, the direct, long-term impact of exogenous GH on melanocytes, skin thickness, and collagen production remains incompletely understood. Therefore, prospective, multi-year, and multicenter studies are essential to track the long-term safety of GH treatment regarding benign (nevi) and malignant (melanoma) skin neoplasm development. Finally, comparative studies, particularly in pediatrics, comparing GH-treated patients with matched controls, are necessary to differentiate the effects of GH from natural growth-related skin changes.

## 6 Conclusion

Growth hormone-related diseases and growth hormone therapy can be associated with skin changes, including both the development of new lesions and exacerbation and/or modifications in existing ones. This article reviews the current state of knowledge regarding skin lesions in patients treated with growth hormone and in growth hormone excess disorders. A variety of skin abnormalities have been reported in diseases such as neurofibromatosis, acromegaly, gigantism, Carney complex, McCune–Albright syndrome, and multiple endocrine neoplasia type 1. It is intriguing that a single hormone, such as growth hormone, can contribute to skin thickening, skin dryness, acrochordons (skin tags), epidermoid cysts, pseudoacanthosis nigricans, seborrheic keratosis, melanocytic nevi, freckling, angiofibromas, collagenomas, lipomas, lentigines, and the growth of hypertrophic scars and keloids. Nonetheless, the exact link between growth hormone and the aforementioned skin lesions is complex and not fully understood. In summary, GH therapy can have a range of effects on the skin, from accelerated nevus growth and skin eruptions to improved wound healing. Understanding these potential effects and monitoring skin health are crucial for patients undergoing GH treatment.
